# Vertebroplasty and Kyphoplasty Can Restore Normal Spine Mechanics following Osteoporotic Vertebral Fracture

**DOI:** 10.4061/2010/729257

**Published:** 2010-06-20

**Authors:** Jin Luo, Michael A. Adams, Patricia Dolan

**Affiliations:** Department of Anatomy, University of Bristol, Southwell Street, Bristol BS2 8EJ, UK

## Abstract

Osteoporotic vertebral fractures often lead to pain and disability. They can be successfully treated, and possibly prevented, by injecting cement into the vertebral body, a procedure known as vertebroplasty. Kyphoplasty is similar, except that an inflatable balloon is used to restore vertebral body height before cement is injected. These techniques are growing rapidly in popularity, and a great deal of recent research, reviewed in this paper, has examined their ability to restore normal mechanical function to fractured vertebrae. Fracture reduces the height and stiffness of a vertebral body, causing the spine to assume a kyphotic deformity, and transferring load bearing to the neural arch. Vertebroplasty and kyphoplasty are equally able to restore vertebral stiffness, and restore load sharing towards normal values, although kyphoplasty is better at restoring vertebral body height. Future research should optimise these techniques to individual patients in order to maximise their beneficial effects, while minimising the problems of cement leakage and adjacent level fracture.

## 1. Introduction

Vertebral fracture is the most common type of osteoporotic fracture and imposes a significant burden on society. In the year 2000, an estimated 1.4 million osteoporotic vertebral fractures were recorded in the world [1]. Such fractures can cause disabling pain and kyphotic deformity [2] leading to impaired physical function and reduced quality of life [3, 4]. For a significant number of patients the pain becomes chronic, even after several months of conservative treatment such as bed rest and analgesics. In recent years, a novel treatment named “vertebroplasty” has been used increasingly to treat painful osteoporotic vertebral fracture [5, 6]. It is a minimally invasive technique that involves injection of bone cement into the fractured vertebral body to stabilize the fracture and alleviate pain. A modification of the technique, called “kyphoplasty”, involves inflating a balloon inside the fractured vertebral body in order to reduce the fracture and create a cavity for the subsequent injection of cement [7]. Kyphoplasty may reduce the incidence of cement leakage during injection [8, 9], and may also help to restore vertebral body height [10–15]. Numerous clinical studies have demonstrated that vertebroplasty is effective in relieving pain following vertebral fracture [16–19]. Furthermore, a recent systematic review of vertebral augmentation for treating vertebral compression fractures suggests that physical disability, general health, and pain relief show greater early improvements in patients treated with verterboplasty or kyphoplasty compared to those undergoing medical management [20]. However, two recent randomized controlled clinical trials found that the pain relief effect of vertebroplasty is no better than local anaesthetic [21, 22]. These controversies suggest that the mechanical and clinical effectiveness of vertebroplasty needs further investigation [23]. 

In this paper, we will concentrate on the mechanical effects of vertebroplasty and kyphoplasty and how they might improve clinical outcome. Although the primary purpose of these procedures is to mechanically augment the fractured vertebral body in order to alleviate pain, the discussion of their mechanical effects should not be limited to their effects on this structure alone. As will be discussed later in this paper, osteoporotic fracture not only damages the fractured vertebral body, but also causes profound changes to the mechanics of the whole spine. It is therefore necessary to take a wider perspective of the mechanical effects of vertebral augmentation. 

This paper will present evidence from current studies to answer the following three questions: (1) What are the effects of osteoporotic vertebral fracture on spine mechanics? (2) To what extent can vertebroplasty and kyphoplasty restore these fracture-induced effects on spine mechanics? (3) What are the important modifiable factors that can influence the restoration effects of vertebral augmentation?

## 2. Osteoporotic Vertebral Fracture Disrupts Spine Mechanics

The main function of the human spine is to resist compressive load in order to maintain the upright posture, allow flexibility for body movements, and protect the spinal cord which lies within the bony vertebral canal [24]. Two main structures of the spine, that is, the vertebrae and intervertebral discs, help to accomplish these functions. The vertebral body has a high stiffness which enables it to resist axial loading, and the intervertebral discs allow for mobility while distributing compressive load to the adjacent vertebral bodies. In a young and uninjured spine more than 80% of the compressive load is transferred through the anterior column (vertebral bodies and discs), and the discs, which act like a water bed, distribute the resulting compressive stress evenly across the vertebral bodies in both flexed and erect postures [25]. 

As the spine ages, osteoporosis and disc degeneration can alter the load bearing properties of the spine. Osteoporosis leads to a loss of stiffness in the bone, and as a result vertebral bodies become more deformable and may show greater deformations than the discs under compressive loading [26]. Disc degeneration leads to a loss of fluid and of disc height. As a result, nucleus pressure falls and the disc loses its ability to distribute compressive stresses evenly on the adjacent vertebral bodies [27]. In flexed postures, stress concentrations develop in the anterior annulus whereas in erect postures, stress becomes concentrated in the posterior annulus and neural arch [27, 28]. These changes in spinal load sharing can lead to stress shielding of the anterior vertebral body in upright postures increasing the risk of osteoporotic vertebral fracture, which can lead to even more profound changes in the spine's mechanical function.

**Figure 1:**
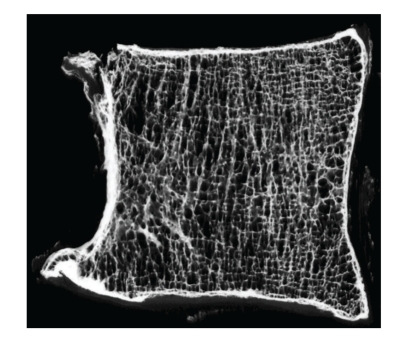
Microradiograph of a mid-sagittal-plane slice of an L2 vertebral body (male, aged 81 years), anterior on the left. Note the inferior trabecular architecture in the anterior region. (Reproduced with permission from Adams et al. [27]).

Osteoporotic vertebral fracture usually involves damage to the endplate, as well as to the trabecular and cortical bone [29]: this leads to a loss of stiffness and strength in the fractured vertebra. Damage is usually located in the anterior part of the vertebral body because this part has lower bone mineral density in elderly spines (Figure 1), and so is easily damaged during spinal flexion when load is concentrated on the anterior part of the disc and vertebral body [27, 29]. This reduces vertebral height anteriorly, leading to wedge shape vertebral deformity [29]. The time-dependent mechanical properties of the fractured vertebra also deteriorate. A recent study on cadaver motion segments found that creep deformation of damaged vertebra was markedly increased following fracture [30], suggesting that the damaged vertebral body may continue to lose height even if no further damage is sustained [30], leading to even more pronounced wedge deformity [31, 32]. 

Vertebral fracture also causes mechanical changes to the surrounding structures. The damaged endplate and trabecular bone deform excessively under compressive load [33] allowing more space for the nucleus of the adjacent disc which is effectively a pressurised fluid [34]. This will induce a loss of intradiscal pressure [29]. A decompressed disc bulges radially and loses height, like a flat tyre [35] producing slack in the intervertebral ligaments, and reducing bending and compressive stiffness [36]. The decrease in nucleus pressure causes more compressive load to be resisted by the annulus. This increases concentrations of stress in the annulus, particularly the posterior annulus [37]. The compressive load resisted by the anterior vertebral body is correspondingly reduced [29, 38]. On the other hand, compressive load bearing by the neural arch is increased significantly because disc height loss brings adjacent vertebrae closer together, increasing contact stresses in the zygapophyseal joints, particularly in erect or extended postures [39]. 

These mechanical changes to adjacent structures following vertebral fracture may have serious consequences. The outer posterior annulus and zygapophyseal joints are innervated, so high stresses in these structures could contribute to the pain associated with osteoporotic vertebral fracture [40, 41]. In the long term, the transfer of compressive load from the anterior vertebral body to the neural arch will stress-shield the entire anterior column, reducing bone density in this region [28]. This could contribute to the risk of adjacent level fracture [27]. The altered disc mechanics such as loss of nuclear pressure and increased stress peaks in the annulus may also initiate or exacerbate disc degeneration [34]. 

Osteoporotic vertebral fracture also influences the mechanics of the whole spine. Increased vertebral wedging at the fractured level would act to increase flexion deformity so that greater extensor moments are required to counter gravitational forces on the trunk and maintain the upright posture. As a result, the compressive forces acting down the spine will increase during standing [42, 43]. This increase in spinal loading may induce anterior wedging at adjacent and other levels with low anterior BMD leading to progressive spinal deformity and loss of sagittal balance [44, 45]. This effect may be exacerbated with time by the marked increase in creep deformation of damaged vertebra [30] which can result in a progressive increase in kyphosis [32]. 

The influence of osteoporotic vertebral fracture is two dimensional: it disrupts the mechanics of the whole spine in space, and this disruption is progressive over time. This poses a serious challenge for its treatment. In the following section, we will present evidence showing how vertebroplasty has the ability to restore spine mechanics in both of these dimensions.

## 3. Vertebroplasty Can Restore Normal Mechanics to an Injured Spine

### 3.1. Stiffness and Strength

Vertebroplasty increases the stiffness and strength of a fractured vertebral body towards prefracture levels [46, 47]. The compressive and bending stiffness of whole spinal “motion segments” (two vertebrae and the intervening disc and ligaments) is also partially restored by vertebroplasty [29]. These effects depend on the type and volume of injected cement, as discussed below.

### 3.2. Height and Wedge Angle

By increasing stiffness, vertebroplasty can effectively increase the height [48–50], and decrease slightly the wedge angle [49], of unloaded fractured vertebrae. Some *in vitro* biomechanical experiments have reported that, if enough cement is injected, then kyphosis angle can be restored to prefracture levels [51]. However, most experimental and clinical studies show that vertebroplasty does not entirely restore height and wedge angle [29, 49, 50, 52–55]. This may reflect the recent tendency to use small cement volumes in order to minimise the risk of leakage, resulting in an insufficient volume of cement being injected. Such a suggestion is supported by the findings of a cadaveric study which found that the restoration of local kyphosis angle was significantly correlated with cement volume [51]. 

Changes in vertebral body shape may be maintained during subsequent loading, although the evidence is equivocal. Augmented vertebral bodies have been reported to show improved fatigue properties compared with nonaugmented controls [56], and several *in vitro* studies have found no loss of restored vertebral height following cyclic loading [51, 57]. Clinical studies found that kyphosis was decreased immediately [54] and 6 months after vertebroplasty [58]. However, more recent clinical studies have noted that augmented vertebral bodies often lose height or recollapse during the follow-up period [59, 60], and in most cases, these changes were not due to trauma [60]. This raises serious concerns about the ability of vertebroplasty to fully and permanently restore height and shape to fractured vertebrae.

### 3.3. Load-Sharing and Adjacent-Level Fracture

**Figure 2:**
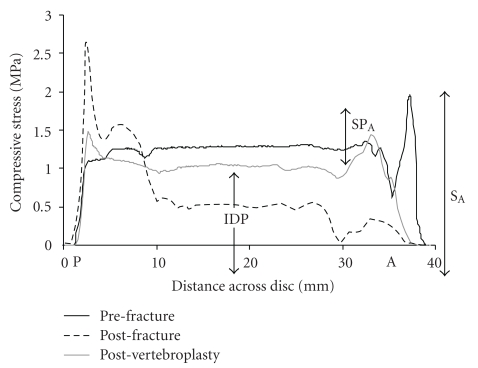
“Stress profiles” show the distribution of compressive stress within the intervertebral disc of a cadaver motion segment (Male 74, L1-2, A: anterior, P: posterior). In the nucleus of the disc, there is a hydrostatic pressure, the intradiscal pressure (IDP). Before fracture, stress is distributed evenly across the disc except for a small stress peak in the anterior annulus (SP_A_). After fracture, IDP falls markedly and a large stress peak appears in the posterior annulus. Vertebroplasty restores IDP towards pre-fracture levels and also reduces the height of the stress peak in the posterior annulus. (Reproduced with permission from Luo et al. [29]).

By augmenting the fractured vertebra, vertebroplasty can help restore normal mechanics to surrounding structures. Endplate deformation of fractured vertebrae under compressive load is reduced after vertebroplasty [61], restoring nucleus pressure in adjacent intervertebral discs, and reducing stress concentrations in the posterior annulus [29, 38]. Compressive load bearing by the anterior half of augmented and adjacent vertebral bodies is also increased, and neural arch load bearing correspondingly decreased [29, 38]. Fracture-induced changes are largely but not entirely reversed (Figure 2). By restoring normal load sharing, vertebroplasty has the potential to decrease the risk of recurrent and adjacent level fractures to an osteoporotic spine.

Despite these findings, there is persisting concern that vertebroplasty can increase the risk of fracture to adjacent vertebrae [62–64] by increasing the compressive stress acting on them [65–68]. Finite element studies suggest that vertebroplasty can increase endplate deformation in adjacent vertebrae by decreasing endplate bulging of the augmented vertebra and thereby increasing intradiscal pressure [65]. However, this is not supported by experimental studies which found that endplate deformation [69] and load transfer [70] do not increase following vertebroplasty.

## 4. Factors Influencing the Mechanical Efficacy of Vertebroplasty

The mechanical effects of vertebroplasty depend on the characteristics of the procedure (such as cement type, volume, and distribution) and also on the characteristics of the augmented spine (including BMD, disc degeneration, and damage severity). 

### 4.1. Properties of Bone Cement

Polymethylmethacrylate (PMMA) is currently the most widely used bone cement for vertebroplasty. However, it has several disadvantages, such as temperature rises during polymerization that can cause tissue damage [71], and lack of bioactivity [72]. Accordingly, new types of cement such as bioactive composite materials like Cortoss and calcium phosphate cement (CPC) have been developed. Although different cements have varying elastic modulus and compressive strength [47, 73], they are all able to increase stiffness and strength of fractured vertebrae. However, this ability depends on the volume injected [47]. 

A finite element study has suggested that stiffer cement can increase stress on the endplates immediately above and below it, leading to increased pressure in adjacent discs, and consequently greater stress on the endplate of adjacent vertebrae [74]. However, this was not confirmed in an experimental study on cadaver motion segments that compared Cortoss and PMMA [29]. Although Cortoss has an elastic modulus twice as high as PMMA [73], no differences were found between the two cements regarding the restoration of intradiscal pressure, spinal load sharing, and compressive and bending stiffness. This could be due to the fact that smaller volumes of Cortoss were used, and it suggests that the mechanical effects of vertebroplasty depend as much on cement volume and distribution as on cement modulus [74]. 

Less stiff bone cements, such as CPC, appear to have inferior fatigue properties as indicated by the appearance of small cracks after cyclic loading [56]. This could explain why clinical studies report that CPC-injected vertebral bodies are vulnerable to progressive collapse for 2 or more years after vertebroplasty [75]. Recently, efforts have been made to reduce the stiffness of PMMA cement for vertebral augmentation [76, 77] but the ability of softer cements to reduce the risk of adjacent-level fracture has yet to be demonstrated [78].

### 4.2. Volume and Distribution of Cement

Experiments on isolated cadaver vertebral bodies show that different volumes of cement are required to restore vertebral strength and stiffness. Strength can be restored to prefracture levels by using as little as 2 ml of PMMA cement [48], but full restoration of vertebral body stiffness requires injection volumes of approximately 4 ml in thoracic vertebrae and 6 to 8 ml in thoracolumbar vertebrae [48, 79, 80]. Restoration of strength and stiffness depends also on percentage volumetric fill [46, 81–83]: 16% [82] to 24% [83] percentage cement fill can fully restore vertebral strength to pre-fracture levels, but 24% [83] to 30% [82] fill is required to restore vertebral stiffness.

**Figure 3:**
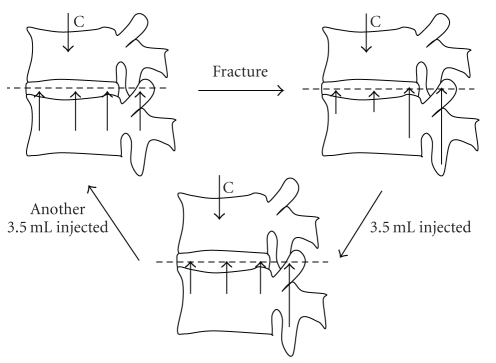
Diagram summarising the changes in load bearing by vertebrae following fracture and vertebroplasty. The length of the upward pointing arrows represents load bearing by different regions of the vertebra. Before fracture (A), the compressive load is borne mostly by the anterior column (disc and vertebral bodies) and stress is distributed evenly across the disc and adjacent vertebral bodies. After fracture (B), stress falls in central and anterior regions of the disc and increases in the posterior annulus and neural arch. Injecting 3.5 ml of cement into the fractured vertebral body (C) causes stress to be distributed more evenly across the disc, but loading on the neural arch remains elevated. Injecting a further 3.5 ml of cement restores neural arch load bearing towards pre-fracture levels (A). Based on data from Luo et al. [84].

The restoration of mechanics to adjacent structures is also influenced by cement volume. One experiment on cadaver motion segments found that only a small amount (3.5 ml) of PMMA is needed to restore normal stress distribution to the fractured and adjacent vertebral bodies, but more cement (7 ml) is required to restore motion segment stiffness and load sharing between the vertebral bodies and neural arches [84], as shown in Figure 3. Restoration of vertebral body shape and kyphosis angle also increases with the cement volume injected [51]. These experimental findings appear to suggest that more cement is beneficial in restoring the spine's mechanical properties. However, a large cement volume may not be advisable clinically, because it increases the risk of cement leakage [85]. Overfilling of the fractured vertebra may also increase the risk of adjacent vertebral fracture [62, 65, 66]. 

This dilemma may be overcome by using larger volumes of more compliant cement, as discussed above. However, there is still a higher risk of cement leakage associated with greater cement volumes. Another solution is to place the bone cement in a more efficient way so that less cement is needed to achieve a better mechanical outcome. For example, placing the cement adjacent to the endplates so that a complete endplate-to-endplate fill pattern is achieved could maximise the increase in compressive stiffness and strength to a fractured vertebral body [74, 86]. Unfortunately, it may also induce excessive endplate deformation in the *adjacent* vertebra and cause adjacent-level fracture [65, 74]. It is therefore reasonable to suggest that a moderate amount of cement placed adjacent to the endplates could restore spine mechanics and minimise the risk of adjacent-level fracture. This suggestion is supported by two recent experimental studies: one showed that the increase in nucleus pressure and the decrease in neural arch load bearing were correlated with cement fill in the region adjacent to the endplate [87]; the other showed that if cement is not in close contact with the endplates then it does not increase endplate deformation in adjacent vertebrae [69]. 

The efficiency of cement placement within the vertebral body can be controlled by cement viscosity during injection. Optimal cement viscosity can result in more evenly distributed cement and can significantly decrease the risk of cement leakage [88, 89]. A more evenly distributed cement pattern results in greater increases in vertebral body stiffness and induces smaller stress concentrations around the cement, which may decrease the risk of adjacent-level fracture [90].

### 4.3. Kyphoplasty versus Vertebroplasty

Kyphoplasty is a modification of the basic vertebroplasty technique. It involves forcibly inflating a balloon inside the fractured vertebral body in order to reduce the fracture and create a cavity for the subsequent injection of cement [91]. This modification is thought to have several benefits: it allows cement to be injected at lower pressure so that leakage is reduced [8, 9, 92], and it leads to compaction of bone around the balloon, elevating the fractured endplate and restoring vertebral body height [10–13, 15, 91, 93–95], which may be beneficial for restoring spine mechanics to patients with osteoporotic fracture [42].

*In vitro* biomechanical studies have shown that kyphoplasty can achieve a better restoration of vertebral height [57, 96, 97] and wedge angle [50] in fractured vertebrae. However, the short-term mechanical effects of kyphoplasty are similar to those of vertebroplasty, with both procedures restoring motion segment stiffness [50, 98], intradiscal pressure [50, 99], and spinal load sharing [50] by a similar amount. A recent randomized clinical trial comparing kyphoplasty and vertebroplasty found that the two procedures produced similar pain-relieving effects, although kyphoplasty was superior in restoring vertebral height and shape [100]. Nevertheless, an in vitro study found that, whilst kyphoplasty achieved a better initial vertebral height restoration than vertebroplasty, the restored height was lost during subsequent cyclic loading [57]. This highlights the importance of following-up changes over time, both *in vivo* and *in vitro*.

### 4.4. Characteristics of the Treated Spine

Cadaver experiments have shown that vertebrae with lower BMD tend to sustain more severe fractures and lose more height [29]. These same specimens show greater changes in mechanical function following fracture [29] and, encouragingly, benefit most from vertebral augmentation [29, 101, 102]. Evidently, vertebroplasty is particularly effective for restoring spine mechanics in patients with osteoporosis.

## 5. Summary and Future Directions

Osteoporotic vertebral fracture can induce profound disruption to normal spine mechanics which can have both short-term and long-term consequences. By augmenting the fractured vertebra, vertebroplasty largely restores normal mechanics to a fractured osteoporotic spine. 

Further research is required to optimise vertebral augmentation procedures. Cadaveric experiments have been successful in identifying the mechanical consequences of fracture, for the affected and adjacent vertebrae, and demonstrating how they can be reversed. However, many variable and interacting factors can influence mechanical outcome, and clinical outcome will depend on even more variables, including the tissue origins of pain. It is becoming evident that mathematical modelling based on patient-specific anatomy and BMD will be required to provide optimal solutions for individual patients. 

In addition, a wider view of vertebral deformity needs to be adopted. Approximately half of patients with osteoporotic vertebral fractures recall no traumatic onset [103], and many deformed vertebrae do not appear to be obviously fractured on radiographs. This suggests that vertebral deformity in many patients involves gradual processes such as “creep”, which is continuing deformation under constant load [30, 104]. Cadaveric studies have recently demonstrated that creep can cause anterior wedge deformities in old human vertebrae bones [104], and that creep is accelerated greatly following vertebral microdamage [30]. Vertebroplasty may prove as successful in modifying these time-dependent processes as in reversing the effects of fracture. 

Finally, more research is required to explain why vertebral deformity is so variably associated with pain, and why pain relief following vertebroplasty is so unpredictable.
